# PIK3C2B drives lung cancer progression through coordinating metabolic reprogramming and EMT-mediated metastasis

**DOI:** 10.1016/j.bbrep.2025.102380

**Published:** 2025-11-21

**Authors:** Xinyue Chou, Wenqian Li, Yandong Li, Zhihan Zhang, Mei Zhong, Hongli Pan, Lili Guo, Fengjie Guo

**Affiliations:** a South China University of Technology of Medicine, Guangzhou, 510006, China; b The Second Affiliated Hospital of Xi'an Medical University, Xi'an, 710038, China; c Tianjin Key Laboratory of Lung Cancer Metastasis and Tumor Microenvironment, Tianjin Lung Cancer Institute, Tianjin Medical University General Hospital, Tianjin, 300052, China; d Laboratory of Molecular Translational Medicine in Chronic Diseases, Shanxi Provincial People's Hospital, Taiyuan, 030012, China

**Keywords:** PIK3C2B, Lung cancer, Metabolic reprogramming, EMT, Mitochondrial flexibility, Prognostic biomarker

## Abstract

Lung cancer is the leading cause of cancer-related mortality worldwide, is driven by metastatic dissemination and metabolic adaptation. However, the underlying mechanisms remain poorly understood. This study identifies PIK3C2B, an understudied isoform of PI3K, as a key regulator of these processes. Integrative transcriptomics analysis of eight GEO datasets revealed 336 consistently dysregulated genes, with PIK3C2B emerging as a candidate associated with metastasis. Elevated expression of PIK3C2B was correlated with reduced overall survival (log-rank p < 0.05) and shorter disease-free survival (p < 0.05) in lung adenocarcinoma patients. Immunohistochemistry confirmed PIK3C2B overexpression in patient tumors. Functional validation showed that silencing PIK3C2B impaired proliferation and migration, while its overexpression enhanced these phenotypes. Mechanistically, PIK3C2B expression was correlated with epithelial-to-mesenchymal transition (EMT) regulators, suggesting its role in mesenchymal transition. Metabolic profiling using Seahorse analysis revealed that PIK3C2B enhanced mitochondrial oxidative phosphorylation (increased basal OCR and ATP production) and glycolytic flux (and fatty acid metabolism). Our findings establish PIK3C2B as a dual-function oncoprotein that promotes lung cancer progression through EMT activation and bioenergetic reprogramming. Its prognostic significance and role in metabolic adaptability highlight PIK3C2B as a promising therapeutic target for disrupting metastasis and enhancing tumor resilience.

## Highlights

• PIK3C2B is a novel metastasis-associated gene in lung cancer identified by cross-dataset transcriptomics.
• High PIK3C2B correlates with poor survival and stage-dependent progression in lung adenocarcinoma.
• PIK3C2B drives proliferation and migration via EMT activation (ZEB1/SNAI1 upregulation).
• PIK3C2B enhances metabolic flexibility by boosting OXPHOS, glycolysis, and fatty acid oxidation.
• Targeting PIK3C2B may disrupt metabolic-EMT crosstalk in aggressive tumors.

## 1 Introduction

Lung cancer remains the leading cause of cancer-related mortality globally, with metastatic dissemination representing a critical barrier to effective treatment [1]. Despite advances in targeted therapies, molecular mechanisms underlying tumor progression and metabolic adaptation in lung cancer remain incompletely understood [2]. The phosphatidylinositol 3-kinase (PI3K) family, known for its roles in cell signaling and metabolism, includes understudied isoforms such as PIK3C2B, which has been implicated in vesicular trafficking and receptor internalization [3,4]. Recent evidence suggests that PI3K signaling intersects with epithelial-mesenchymal transition (EMT) pathways to promote metastasis, while metabolic reprogramming—a hallmark of cancer—enables tumor cells to meet the bioenergetic demands of proliferation and invasion [5]. However, the functional role of PIK3C2B in coordinating these processes remains unexplored.

In this study, we employed integrative transcriptomics approaches to identify PIK3C2B as a novel metastasis-associated gene in lung cancer. By conducting a cross-dataset analysis of publicly available Gene Expression Omnibus (GEO) repositories, followed by functional validation in vitro and comprehensive metabolic profiling, we demonstrate that PIK3C2B drives tumor progression by enhancing EMT and mitochondrial bioenergetic flexibility. Our findings establish PIK3C2B as a prognostic biomarker and a potential metabolic target in lung cancer.

## 2 Materials and methods

### 2.1 Data acquisition and DEG analysis

Eight lung cancer Gene Expression Omnibus (GEO) datasets (GSE117049, GSE17599, GSE18842, GSE32867, GSE33532, GSE5364, GSE66759, GSE74706) were utilized. The GEO2 tool from the National Center for Biotechnology Information (NCBI) were employed to analyze all datasets included in our study. Differentially expressed genes (DEGs) were identified with p-value <0.05 and |log2 fold change| >1. Cross-dataset intersections were visualized via a Venn diagram (jvenn).

### 2.2 Functional enrichment and survival analysis

Gene Ontology (GO) and KEGG pathway enrichment analyses were conducted using DAVID v6.8, considering a significance threshold of p-value <0.05. The prognostic significance of candidate genes was assessed using Kaplan-Meier survival analysis (GEPIA2), with statistical significance evaluated by log-rank tests.

### 2.3 Immunohistochemistry

Formalin-fixed, paraffin-embedded (FFPE) primary lung adenocarcinoma tissues and matched adjacent normal tissues were sectioned at 4 μm thickness. Deparaffinization and rehydration were performed using xylene and graded ethanol series. Antigen retrieval was conducted in citrate buffer (pH 6.0) at 95 °C for 20 min. Endogenous peroxidase activity was quenched with 3 % hydrogen peroxide for 10 min. Sections were blocked with 5 % normal goat serum for 1 h at room temperature and incubated overnight at 4 °C with anti-PIK3C2B primary antibody (1:200 dilution, Proteintech, 24788-1-AP). After washing, sections were incubated with HRP-conjugated secondary antibody (1:500, Dako, K4001) for 1 h at room temperature. Signal detection was performed using DAB substrate (DakoDako, K3468), followed by counterstaining with hematoxylin. Slides were dehydrated, mounted, and imaged.

### 2.4 Cell culture and cell transfection

A549 and H1299 lung adenocarcinoma cells (ATCC) were separately maintained in DMEM and RPMI-1640 with 10 % FBS. For cell transfection, cells were transfected at 70 % confluence and transfected using Lipofectamine 3000 (Invitrogen, L3000001). PIK3C2B knockdown was achieved with three independent siRNAs targeting PIK3C2B (siPIK3C2B_1, siPIK3C2B_2, siPIK3C2B_3) and a non-targeting scramble siRNA (siCtrl). Overexpression of PIK3C2B was performed using the pCMV-PIK3C2B plasmid, with the empty pCMV vector serving as a control. Transfected cells were harvested 48 h post-transfection for subsequent functional assays.

### 2.5 Real time PCR

Total RNA was extracted from cultured cells using TRIzol reagent (Invitrogen, 15596026) and quantified via Nanodrop spectrophotometry. cDNA synthesis was performed with 1 μg RNA using the PrimeScript RT Reagent Kit (Takara Bio, RR037A) according to the manufacturer's protocol. Real-time PCR amplification was carried out in triplicate using SYBR Green Master Mix (Roche). Gene-specific primers for PIK3C2B (forward: 5′-TCAGGGCAATGGGGAACAC-3′; reverse: 5′-CGTAACAGCTTGAGGTCGGTC -3′) and the housekeeping gene GAPDH (forward: 5′-GGAGCGAGATCCCTCCAAAAT-3′; reverse: 5′-GGCTGTTGTCATACTTCTCATGG-3′) were used. Cycling conditions included an initial denaturation at 95 °C for 10 min, followed by 40 cycles of 95 °C for 15 s and 60 °C for 1 min. Relative expression was calculated using the 2^(-ΔΔCt) method, normalized to GAPDH.

### 2.6 Western blot

Cells were lysed in RIPA buffer (Beyotime, P0013B) supplemented with protease inhibitors (Roche, 04693132001). Protein concentrations were determined via BCA assay (Thermo Fisher, 23225), and 20 μg of total protein per sample was resolved on 10 % SDS-PAGE gels. Proteins were transferred to PVDF membranes (Millipore, IPVH00010), blocked with 5 % non-fat milk in TBST for 1 h, and incubated overnight at 4 °C with primary antibodies: anti-PIK3C2B (1:1000, Proteintech, 24788-1-AP) and anti-GAPDH (1:5000, Santa Cruz Biotechnology, sc-47724). Membranes were washed and incubated with HRP-conjugated secondary antibodies (1:5000, Cell Signaling Technology, 7074) for 1 h at room temperature. Protein bands were visualized using ECL substrate (Millipore, WBKLS0100).

### 2.7 CCK-8 assays

Cell proliferation was assessed using the Cell Counting Kit-8 (Dojindo, CK04). Cells were seeded in 96-well plates (3 × 10^3^ cells/well) and transfected with siRNA or overexpression constructs. At 24, 48, and 72 h post-transfection, 10 μL CCK-8 reagent was added to each well, followed by incubation at 37 °C for 2 h. Absorbance at 450 nm was measured using a microplate reader (BioTek Synergy H1).

### 2.8 Wound healing assays

Cells were seeded in 6-well plates and grown to 90 % confluence. Linear scratches were created using a sterile 200 μL pipette tip, and detached cells were removed by PBS washing. Cells were maintained in serum-free medium to minimize proliferation bias. Wound closure was monitored at 0, 24, and 48 h. Images were analyzed with ImageJ software, and migration rates were calculated as the distance of migration relative to the initial scratch.

### 2.9 Metabolic profiling

Mitochondrial respiration and glycolysis were quantified using a Seahorse XFe96 Analyzer (Agilent). Cells were subjected to sequential treatment with oligomycin (1 μM), Rot/AA (0.5 μM each), 2-DG (10 mM), and FCCP (1 μM). OCR and PER were normalized to cell count.

### 2.10 Statistical analysis

Data are presented as mean ± SEM. Statistical analyses, including Student's t-test, ANOVA, and Spearman correlation, were performed using GraphPad Prism 9. A p-value <0.05 was considered statistically significant.

## 3 Results

### 3.1 Integrative cross-dataset analysis reveals metastasis-associated gene signatures in lung cancer

**Fig. 1:**
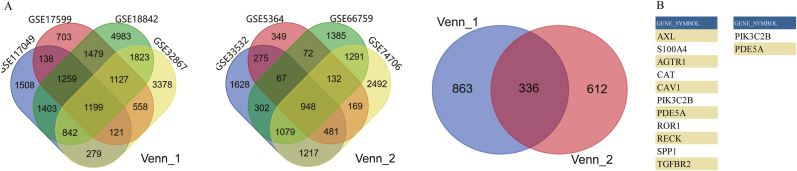
Integrative transcriptomics identifies PIK3C2B as a metastasis-associated gene in lung cancer. (A) Venn diagram illustrating the intersection of consistently dysregulated genes from two independent meta-analyses(Venn_1: GSE117049, GSE17599, GSE18842, GSE32867; Venn_2: GSE33532, GSE5364, GSE66759, GSE74706). The analysis identified 336 genes common across all eight datasets. (B) Bar graph showing selected significantly enriched Gene Ontology (GO) biological processes related to metastasis for the 336 conserved DEGs, as analyzed by DAVID (p < 0.05). PIK3C2B and PDE5A, two candidates with uncharacterized roles in cell migration, are highlighted.

In this study, we conducted an integrative analysis of publicly available GEO datasets to investigate molecular mechanisms underlying lung cancer progression. Eight independent lung cancer-related GEO datasets (GSE117049, GSE17599, GSE18842, GSE32867, GSE33532, GSE5364, GSE66759, GSE74706) were systematically integrated. Statistically significant differentially expressed genes (DEGs) were identified using standardized criteria (adjusted p-value <0.05, |log2 fold change| >1) (Supplementary Fig. 1). A cross-dataset intersection analysis, presented through a Venn diagram, revealed 336 genes that were consistently dysregulated across all examined cohorts (Fig. 1A).

These conserved DEGs were subjected to functional annotation and pathway enrichment analysis using DAVID, which highlighted significant enrichment in biological processes related to neoplasm metastasis (p-value <0.05). Key metastasis-associated genes identified in this analysis included AXL, S100A4, AGTR1, CAT, CAV1, PIK3C2B, PDE5A, ROR1, RECK, SPP1, and TGFBR2 [[6], [7], [8], [9], [10]]. To prioritize candidate genes with unclear roles in cell migration pathways, we focused on PIK3C2B and PDE5A (Fig. 1B).

### 3.2 Clin*ical implications of PIK3C2B as a prognostic biomarker in lung cancer progression*

**Fig. 2:**
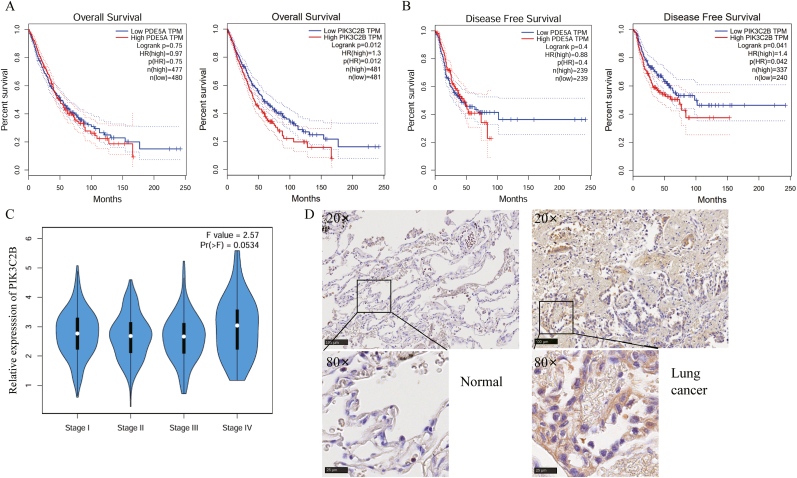
PIK3C2B is a prognostic biomarker in lung adenocarcinoma. (A, B) Kaplan-Meier survival curves for (A) overall survival and (B) disease-free survival of lung cancer patients from the TCGA database, stratified by high (red) and low (blue) PIK3C2B mRNA expression. Statistical significance was determined by the log-rank test (p < 0.05). (C) Box plot showing PIK3C2B mRNA levels across different pathological stages (Stage I-IV) of lung adenocarcinoma from the TCGA database. Data are presented as median with interquartile range; p-value was calculated by one-way ANOVA. (D) Representative immunohistochemistry (IHC) images of PIK3C2B protein expression in primary lung cancer tissues and matched adjacent normal lung tissues. scale bar = 100 μm (20 × ), 25 μm (80 × ).

To assess the prognostic relevance of two candidate genes in lung cancer, we performed comprehensive survival analyses. Kaplan-Meier survival curves generated using data from the GEPIA database revealed that elevated PIK3C2B expression significantly correlated with reduced overall survival (log-rank p < 0.05; Fig. 2A). Moreover, patients with high PIK3C2B expression demonstrated markedly shorter disease-free survival compared to those with low expression (log-rank p < 0.05; Fig. 2B). Stratification by pathological staging showed progressive upregulation of PIK3C2B mRNA levels across advancing tumor stages (Fig. 2C), suggesting stage-dependent accumulation of this molecular marker. In line with these findings, immunohistochemical analysis confirmed pronounced PIK3C2B protein overexpression in primary lung adenocarcinoma specimens relative to matched adjacent normal tissues (Fig. 2D), further supporting its potential role in tumor pathogenesis.

### 3.3 PIK3C2B drives lung cancer cell proliferation and migration via EMT

**Fig. 3:**
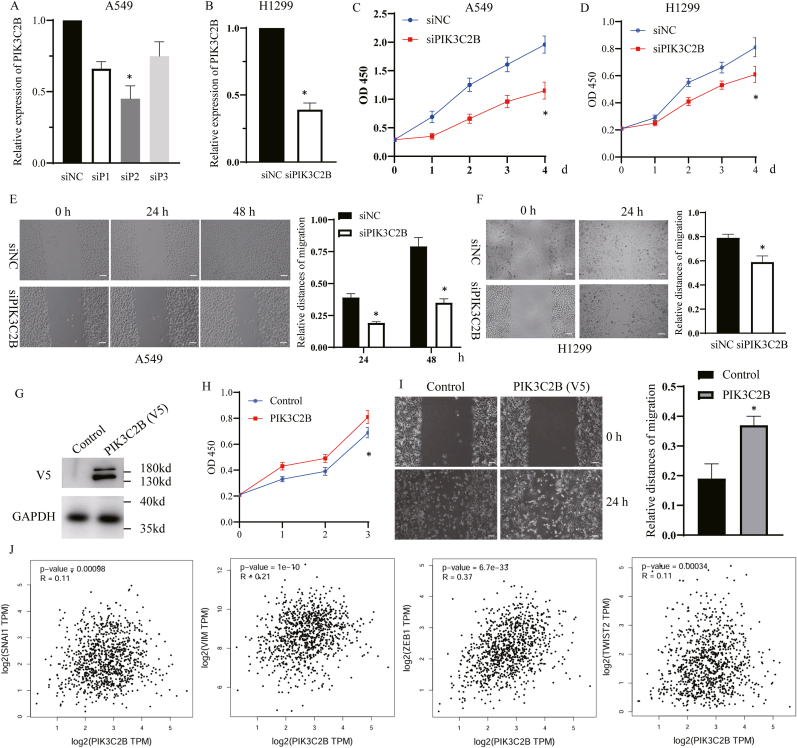
PIK3C2B drives lung cancer cell proliferation and migration via EMT activation. (A, B) Validation of PIK3C2B knockdown efficiency by qPCR in (A) A549 and (B) H1299 lung cancer cells transfected with three independent siRNAs (siP1, siP2, siP3) or a non-targeting scramble control (siNC). Data are normalized to GAPDH and presented as mean ± SEM of triplicate measurements. (C, D) Cell proliferation measured by CCK-8 assays in (C) A549 and (D) H1299 cells following PIK3C2B knockdown or siNC control over 96 h. Data are presented as mean absorbance ± SEM (n = 3). ∗p < 0.05 vs. siNC. (E, F) Wound healing assays in (E) A549 and (F) H1299 cells after PIK3C2B knockdown (∗p < 0.05). Left: Representative bright-field images of the wound at 0 and 48 h. Right: Quantification of the relative wound closure percentage. Data are mean ± SEM (n = 3). ∗p < 0.05 vs. siNC. Scale bar = 100 μm. (G) Detection of PIK3C2B level in lung cancer cells by Western blot. (H, I) Functional consequences of PIK3C2B overexpression. (H) CCK-8 proliferation assay and (I) wound healing migration assay in cells transfected with PIK3C2B overexpressing plasmid or empty vector (Control) Data are mean ± SEM. ∗p < 0.05 vs. control. (J) Correlation of PIK3C2B mRNA expression with key epithelial-mesenchymal transition (EMT) regulator genes (SNAI1, VIM, ZEB1, TWIST2) in the TCGA lung cancer cohort (Spearman's r; p < 0.001). scale bar = 100 μm.

To investigate the functional role of PIK3C2B in lung cancer progression, we utilized RNA interference to silence PIK3C2B in lung cancer cell lines. Screening of multiple siRNA constructs identified siPIK3C2B_2 (siP2) as the most effective silencing construct (Fig. 3A and B, Supplementary Fig. 2). Functional characterization revealed that PIK3C2B depletion significantly impaired cell proliferation, as evidenced by a marked reduction in cell viability at 72 h post-transfection, as measured by CCK-8 assays (p < 0.05, Fig. 3C and D). Wound healing assays demonstrated that PIK3C2B silencing reduced A549 cell migration capacity relative to scramble controls (p < 0.05, Fig. 3E and F), suggesting its critical role in both growth and metastatic potential.

Consistent with these observations, we confirmed PIK3C2B expression in the indicated lung cancer cell line through Western blot analysis (Fig. 3G). Complementary overexpression experiments showed that PIK3C2B amplification enhanced proliferation rates (p < 0.05) and increased migration capacity compared to empty vector controls (Fig. 3H and I). Tumor formation assays in mice using control and PIK3C2B-knockdown Lewis mouse cells showed that PIK3C2B depletion significantly reduces tumor growth in vivo (Supplementary Fig. 3). Moreover, rescue experiments by re-expressing PI3KC2B in PI3KC2B-knockdown cells significantly restored the impaired phenotypes, including cell proliferation and migration, confirming the specificity of the observed effects (Supplementary Fig. 4).

Given the lipid kinase activity of PIK3C2B, we wondered if this activity influenced its function. The results showed that PIK3C2B-D1213A did not promote cell proliferation and migration, indicating the crucial role of PIK3C2B's lipid kinase activity ((Supplementary Fig. 4).

To elucidate the mechanism underlying PIK3C2B-mediated migration, we analyzed epithelial-mesenchymal transition (EMT) markers using the GEPIA database. PIK3C2B expression showed significant positive correlations with key EMT regulators: SNAI1 (r = 0.11, p = 0.00098), VIM (r = 0.21, p = 1e-10), ZEB1 (r = 0.37, p = 6.7e-33), and TWIST2 (r = 0.11, p = 0.00034) in TCGA lung adenocarcinoma samples (Fig. 3J). Morever, PCR further confimed the association in PIK3C2B-knocked-down lung cancer cells (Supplementary Fig. 5). These data suggest that PIK3C2B may facilitate cancer cell migration through EMT.

### 3.4 PIK3C2B enhances mitochondrial metabolic flexibility

Cellular metabolism is essential for maintaining energy balance during growth and migration [11]. To investigate the metabolic reprogramming associated with PIK3C2B in lung cancer cells, we employed the Seahorse extracellular flux analyzer to measure bioenergetic parameters. Oxygen consumption rate (OCR), reflecting oxidative phosphorylation (OXPHOS), and proton efflux rate (PER), reflecting glycolytic flux, were assessed under basal conditions and following sequential pharmacological perturbations: oligomycin (ATP synthase inhibition), rotenone/antimycin A (Rot/AA; electron transport chain [ETC] complex I/III inhibition), 2-deoxyglucose (2-DG; glycolysis blockade), and FCCP (mitochondrial uncoupling).

**Fig. 4:**
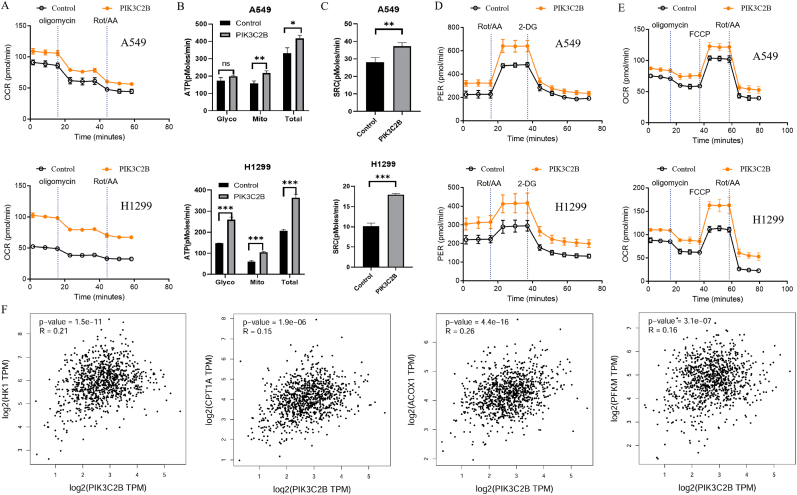
PIK3C2B enhances mitochondrial metabolic flexibility in lung cancer cells. (A–E) Metabolic parameters were assessed using the Seahorse XFe96 Analyzer in control and PIK3C2B-overexpressing cells. (A) Basal oxygen consumption rate (OCR). (B) ATP-linked OCR, calculated as the oligomycin-sensitive respiration. (C) Spare respiratory capacity (SRC), calculated as the difference between maximal OCR (induced by FCCP) and basal OCR. (D) Glycolytic activity represented by the proton efflux rate (PER) under baseline conditions and after inhibition of mitochondrial respiration with rotenone and antimycin A (Rot/AA). (E) Maximal OCR following the addition of the uncoupler FCCP. All metabolic data are normalized to cell count and presented as mean ± SEM of three independent experiments. ∗p < 0.05, ∗∗p < 0.01, ∗∗∗p < 0.001 vs. control (Student's t-test or two-way ANOVA). (F) Network diagram illustrating the significant positive correlations (Spearman's r, all p < 0.01) between PIK3C2B expression and key metabolic enzymes in the TCGA lung cancer cohort.

Mitochondrial ATP production was calculated from oligomycin-sensitive OCR. PIK3C2B-overexpressing cells exhibited significantly elevated basal OCR and mitochondrial ATP synthesis compared to controls, indicating enhanced OXPHOS capacity (Fig. 4A and B). PIK3C2B also enhanced spare respiratory capacity (SRC), quantified by OCR not coupled to ATP production, thereby reflecting mitochondrial metabolic potential (Fig. 4C). Total glycolytic activity, assessed via Rot/AA-induced PER suppression, revealed that PIK3C2B overexpression increased both glycolytic and oxidative energy production (Fig. 4D). Mitochondrial stress testing using FCCP demonstrated greater maximal respiratory capacity in PIK3C2B-expressing cells, suggesting improved metabolic flexibility under energetic demand (Fig. 4E).

Gene expression analysis revealed significant positive correlations between PIK3C2B and key metabolic regulators, including hexokinase 1 (HK1, glycolysis), carnitine palmitoyltransferase 1A (CPT1A, fatty acid oxidation), acyl-CoA oxidase 1 (ACOX1, peroxisomal β-oxidation), and phosphofructokinase muscle isoform (PFKM, glycolysis) (Fig. 4F). PCR also illustrated the association in PIK3C2B-knocked-down lung cancer cells (Supplementary Fig. 6). These findings demonstrate that PIK3C2B promotes a hypermetabolic state by coordinately enhancing mitochondrial oxidative capacity, glycolytic reserve, and expression of critical metabolic enzymes, thereby increasing bioenergetic adaptability in lung cancer cells.

Although the primary focus of our study is on the roles of PIK3C2B in EMT and metabolic reprogramming, we have now explored its potential involvement in immune modulation (Supplementary Fig. 7) and cancer stemness (Supplementary Fig. 8) through gene expression analysis of public datasets.

## 4 Discussion

This study identifies PIK3C2B as a pivotal regulator of lung cancer progression, bridging metastatic dissemination and metabolic adaptation—a nexus that remains underexplored in oncology. While the PI3K/AKT/mTOR axis is well-characterized in cancer metabolism and EMT, most studies focus on canonical isoforms like PIK3CA or PIK3CB. Our findings position PIK3C2B, a class II PI3K, as a unique molecular orchestrator of both processes, expanding the functional repertoire of this kinase family. Recent studies have begun to elucidate the unique roles of class II PI3Ks in cancer; for instance, PIK3C2A has been implicated in sustaining mitogenic signaling and cell division, while PIK3C2G is linked to cytoskeletal reorganization and membrane trafficking [[12], [13], [14]].

The observed correlation between PIK3C2B and EMT regulators (SNAI1, VIM, ZEB1, TWIST2) aligns with previous reports linking PI3K signaling to mesenchymal transition. For instance, PIK3CA mutations drive migration and EMT transition in triple-negative breast cancer (TNBC) [15]. However, our work reveals that PIK3C2B, unlike other isoforms, directly correlates with ZEB1—a master EMT transcription factor implicated in chemoresistance [16]. This parallels recent findings in patients with metastatic colorectal cancer, where PIK3C2B mutations exhibited adverse outcomes [17], suggesting a conserved role across malignancies. Notably, our demonstration of PIK3C2B's pro-migratory effects in lung adenocarcinoma cells complements emerging evidence that class II PI3Ks regulate membrane remodeling [18,19], though this mechanism warrants further exploration.

The dual enhancement of OXPHOS and glycolysis by PIK3C2B challenges the classical “Warburg effect” paradigm, where cancers prioritize glycolysis even under aerobic conditions. Instead, our metabolic profiling supports the “metabolic flexibility” model observed in aggressive tumors [20]. The upregulation of CPT1A and ACOX1 indicates PIK3C2B promotes fatty acid oxidation—a pathway increasingly linked to metastasis [21]. This aligns with studies showing PI3K-dependent large lipid droplet accumulation in Drosophila ovarian nurse cells [22], but contrasts with PIK3CA-mutant tumors that predominantly upregulate glycolysis [23]. Such isoform-specific metabolic roles underscore the need for targeted therapeutic strategies.

Clinically, our survival data corroborate prior cancer analyses identifying PIK3C2B amplification as a prognostic marker [24]. However, its stage-dependent overexpression in lung adenocarcinoma—a finding not reported in other cancers—highlights tissue-specific regulatory mechanisms. While current PI3K inhibitors (e.g., alpelisib) target class I isoforms, our work suggests dual inhibition of PIK3C2B and mTOR (a downstream effector) may synergistically disrupt metabolic-EMT crosstalk. This approach could address limitations of single-pathway targeting, as resistance often arises from compensatory metabolic adaptations [25].

Although our in vitro models establish causality, the lack of in vivo validation (e.g., xenograft metastasis assays) limits translational impact of our findings. Additionally, while we link PIK3C2B to EMT markers, chromatin immunoprecipitation (ChIP) or co-immunoprecipitation experiments are needed to determine whether it directly regulates ZEB1 or SNAI1. This functional profile starkly contrasts with the well-characterized class I PI3K isoform PIK3CA, which predominantly enhances glycolytic flux via AKT activation and often exhibits a stronger association with proliferative signals rather than the coordinated EMT-metabolic plasticity observed here with PIK3C2B [26,27]. Recent work showing PI3Ks modulate HIF-1α stability [28] raises the possibility of hypoxia-driven PIK3C2B activation—a hypothesis testable in 3D spheroid models.

Interestingly, this pro-tumorigenic role of PIK3C2B presents a striking contrast to its recently identified function in neurological disorders, where loss-of-function variants cause focal epilepsy through impaired PI(3,4)P2 synthesis and consequent mTORC1 hyperactivation [29]. This functional dichotomy—where PIK3C2B overexpression drives cancer progression while its deficiency promotes neuronal hyperexcitability—highlights the context-dependent nature of PI3K–C2β signaling in human diseases.

There are sitll some limitations. A key outstanding question is whether the observed migration defect stems directly from PIK3C2B-mediated metabolic reprogramming or from its potential role in regulating focal adhesion/integrin dynamics. The direct impact of PIK3C2B on its lipid products, PI3P and PI3,4P2, remains to be quantified. Future work will precisely measure these phosphoinositides to fully elucidate the lipid signaling mechanism underlying PIK3C2B-driven metabolic and metastatic reprogramming. Future work alos valuate the correlative nature of the association between PIK3C2B and key EMT transcription factors.

## 5 Conclusion

While our findings illuminate a correlation between PIK3C2B expression, metabolic flexibility, and EMT, it is important to frame these observations as preliminary evidence suggesting PIK3C2B's potential role in lung cancer progression. The current data do not establish a definitive causal mechanism. Future studies are essential to validate these associations and determine whether the coordinated regulation of metabolism and EMT is a direct function of PIK3C2B or a downstream consequence of broader signaling network alterations.

## Ethics approval and consent to participate

This study protocol was approved by the Medical Ethics Committee of Tianjin Medical University General Hospital (IRB-2020-KY-101) (March 26, 2020) and followed the Declaration of Helsinki.

## CRediT authorship contribution statement

**Xinyue Chou:** Data curation, Investigation, Methodology. **Wenqian Li:** Formal analysis, Investigation, Methodology. **Yandong Li:** Formal analysis, Investigation, Methodology. **Zhihan Zhang:** Formal analysis, Investigation, Methodology. **Mei Zhong:** Investigation, Methodology. **Hongli Pan:** Investigation, Methodology, Supervision. **Lili Guo:** Data curation, Formal analysis, Resources, Supervision, Writing – review & editing. **Fengjie Guo:** Conceptualization, Data curation, Funding acquisition, Project administration, Resources, Supervision, Writing – original draft, Writing – review & editing.

## Declaration of competing interest

The authors have no conflicts of interest.

## Data Availability

No data was used for the research described in the article.
