# Supplementary material for: PIK3C2B drives lung cancer progression through coordinating metabolic reprogramming and EMT-mediated metastasis
Source: Biochem Biophys Rep. 2025 Nov 21;44:102380. doi: 10.1016/j.bbrep.2025.102380 (PMC12681860; doi:10.1016/j.bbrep.2025.102380)
Supplement: Multimedia component 1 [file mmc1.docx]

**Supplementary Methods**

**Xenograft Tumor Formation Assay**

Female C57BL/6 mice (6-8 weeks old) were housed under specific pathogen-free conditions. Lewis lung carcinoma (LLC) cells stably expressing control or PIK3C2B-specific shRNA were harvested and resuspended in PBS. A total of 1×10^6^ cells in 100 µL were subcutaneously injected into the right flank of each mouse (n = 3 per group). Tumor dimensions were measured every 4 days using a caliper, and tumor volume was calculated using the formula: V=0.5×length×width^2^. Mice were euthanized 23 days post-injection, and tumors were excised and weighed.

**Rescue and Kinase-Dead Mutant Experiments**

For rescue experiments, PIK3C2B-knockdown cells were transfected with a human PIK3C2B expression plasmid (pCMV-PIK3C2B) using Lipofectamine 3000 (Invitrogen). To evaluate the role of lipid kinase activity, the kinase-dead mutant PIK3C2B-D1213A was generated by site-directed mutagenesis and transfected into lung cancer cells. Transfected cells were subjected to functional assays post-transfection.

**Analysis of Immune Cell Infiltration**

The correlation between PIK3C2B expression and immune cell infiltration levels in lung adenocarcinoma was analyzed using the TIMER2.0 web server ([http://timer.cistrome.org/](http://timer.cistrome.org/)). Spearman correlation analysis was performed to assess the association of PIK3C2B expression with the abundance of M2 macrophages, regulatory T cells (Tregs), and CD8+ T cells in the TCGA-LUAD dataset.

**Cancer Stemness Marker Correlation Analysis**

The correlation between PIK3C2B expression and established lung cancer stemness markers (CD44, ALDH1A1, POU5F1/OCT4) was evaluated using the GEPIA2 database ([http://gepia2.cancer-pku.cn/](http://gepia2.cancer-pku.cn/)). Spearman correlation analysis was performed using transcriptomic data from the TCGA-LUAD dataset.

**Quantitative PCR Analysis for EMT and Metabolic Markers**

Total RNA was extracted from control and PIK3C2B-knockdown A549 and H1299 cells using TRIzol reagent (Invitrogen). cDNA was synthesized using the PrimeScript RT Reagent Kit (Takara Bio). Quantitative PCR was performed with SYBR Green Master Mix (Roche) using gene-specific primers for EMT markers (SNAI1, VIM, ZEB1, TWIST2) and metabolic genes (CPT1A, ACOX1, HK1). GAPDH was used as an internal control. Relative expression was calculated using the 2^−ΔΔCt^​method.

**Supplementary Figures**


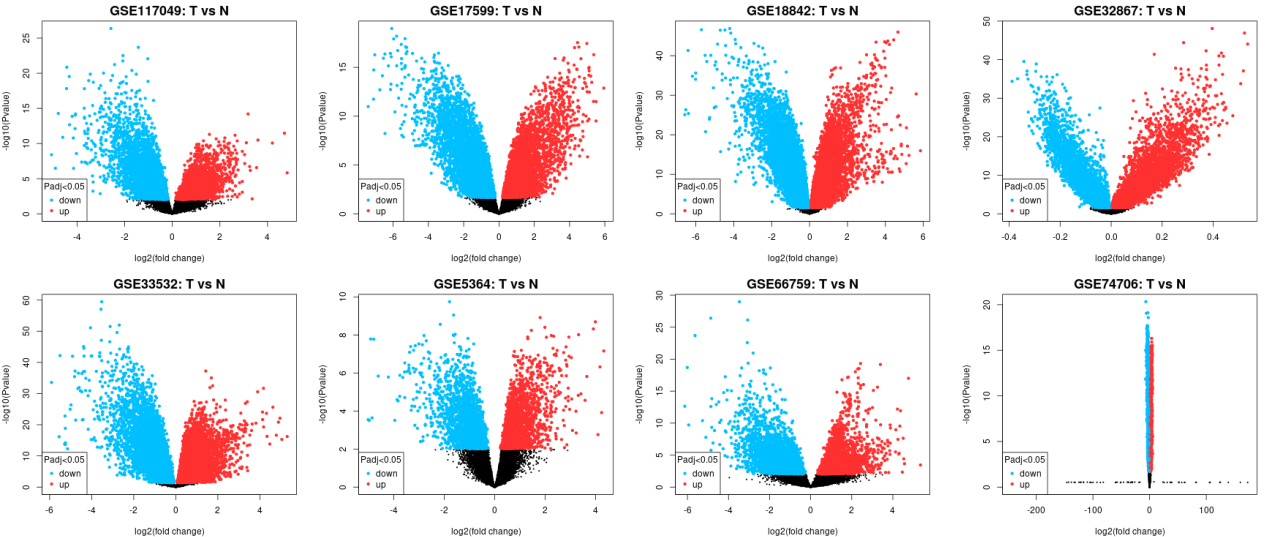


**Supplementary Figure 1**. Volcano Plots displaying differentially expressed genes (DEGs) in eight independent lung cancer GEO datasets. DEGs were defined by an adjusted p-value < 0.05 and |log2 fold change| > 1. Significantly upregulated and downregulated genes are highlighted in red and blue, respectively.


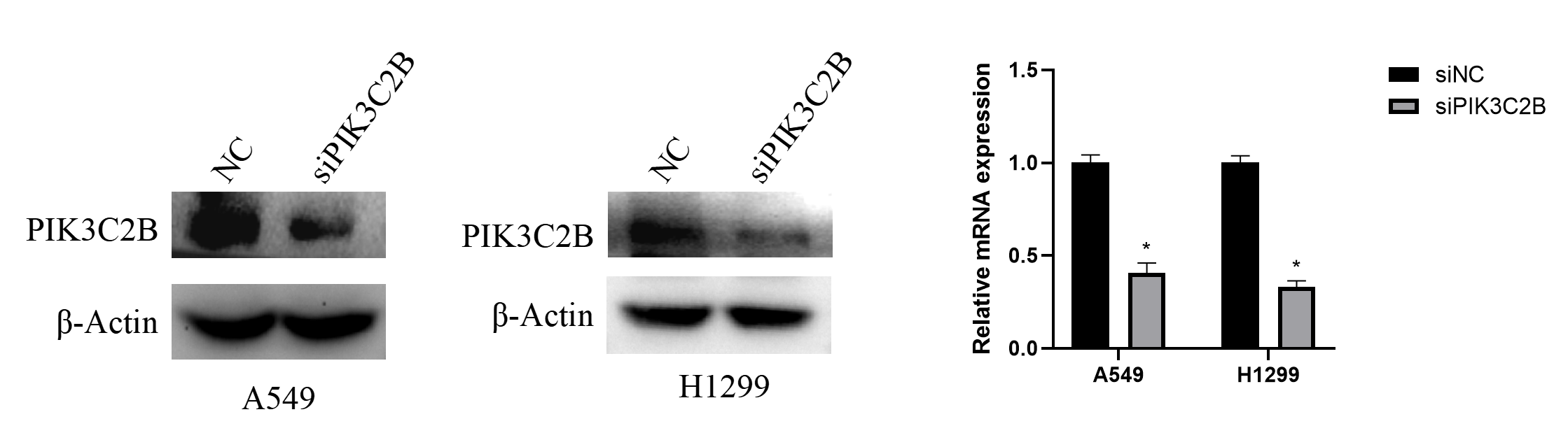


**Supplementary Figure 2**. Protein level of PIK3C2B expression in A549 and H1299 cell lines after PIK3C2B knockdown, detected by Western blot. *p < 0.05.


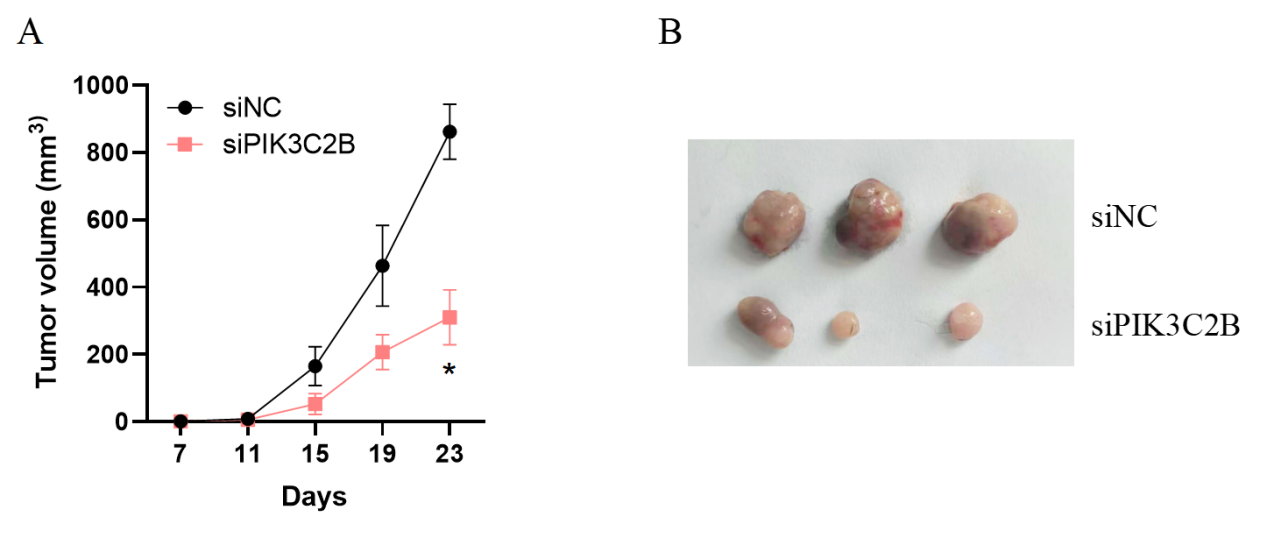


**Supplementary Figure 3**. Xenograft tumor formation assays in mice using control and PIK3C2B-knockdown Lewis lung carcinoma (LLC) cells. Tumor volume was measured, and mice were sacrificed after 23 days to isolate tumors. (A) Tumor growth curves showed significant suppression in the PIK3C2B-knockdown group compared to the controls. (B) Representative images of excised tumors highlight the reduced tumor size upon PIK3C2B depletion. *p < 0.05.


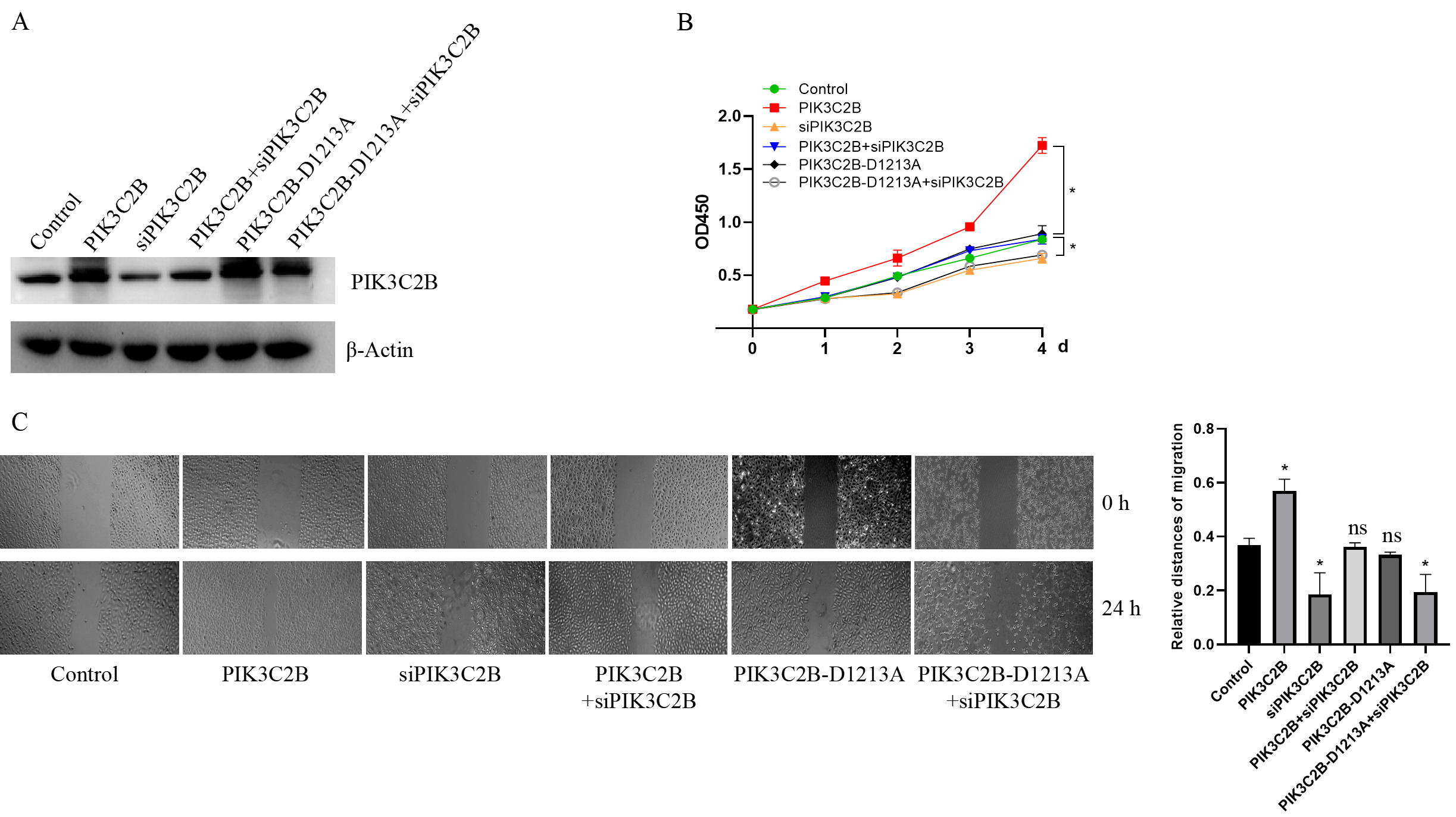


**Supplementary Figure 4**. Cell proliferation and migration analysis. (A) The protein level of PIK3C2B was detected by western blot. (B) Cell proliferation was investigated after re-expression of PIK3C2B in PI3KC2B-knockdown cells or expression of the PI3KC2B mutant (PIK3C2B-D1213A) in the lung cancer cells. (C) Cell migration was assessed using a wound healing assay in lung cancer cells upon re-expression of PIK3C2B or its D1213A mutant. Images were captured at 0 and 24 hours post-wounding. *p < 0.05, compared with the control group. Data are representative of three independent experiments, and error bars indicate mean ± SEM.


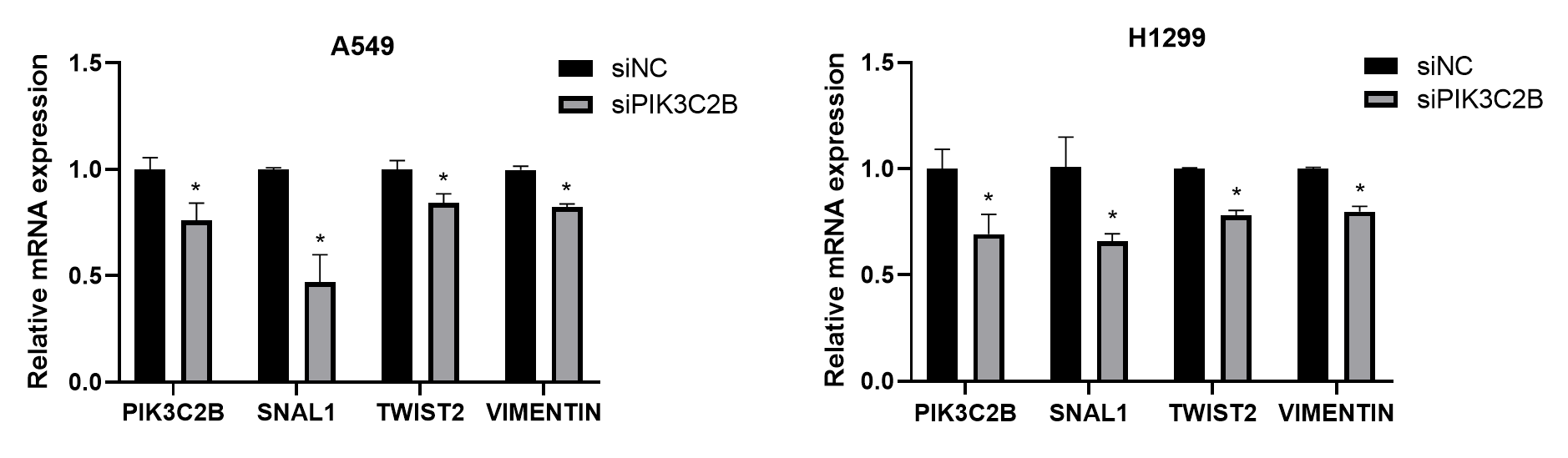


**Supplementary Figure 5**. The levels of EMT transcription factors were measured by qPCR in lung cancer cell lines (A549 and H1299) following the knockdown of PIK3C2B. *p < 0.05.


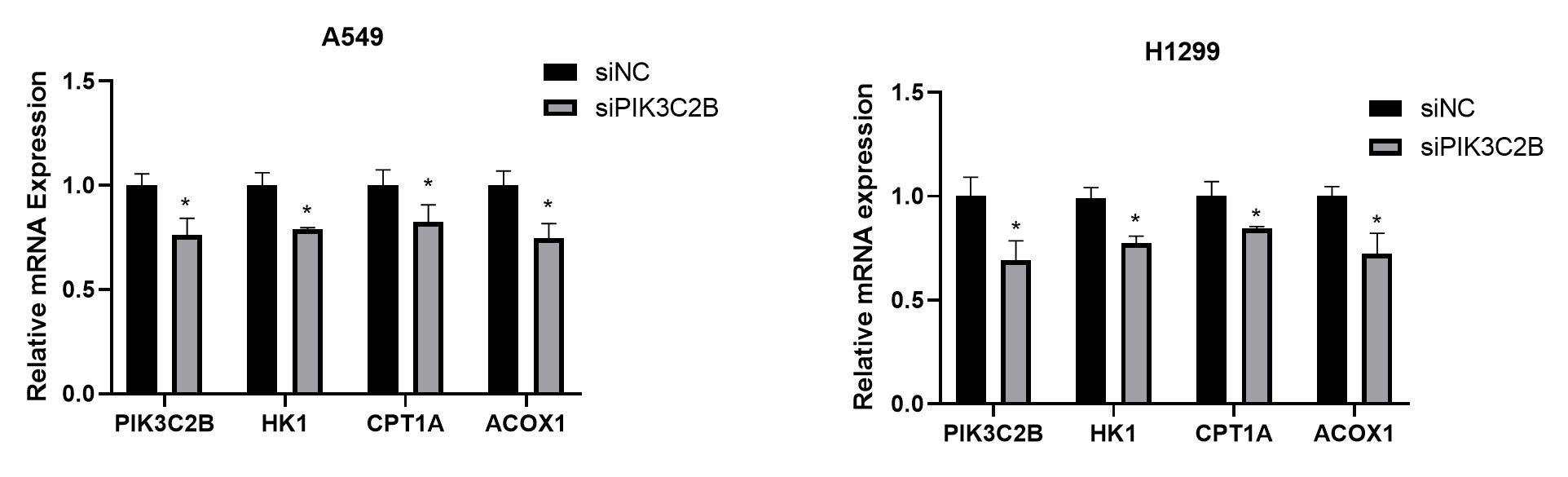


**Supplementary Figure 6**. After the knockdown of PIK3C2B, the levels of CPT1A, ACOX1, and HK1 were assessed by qPCR in lung cancer cell lines (A549 and H1299). *p < 0.05.


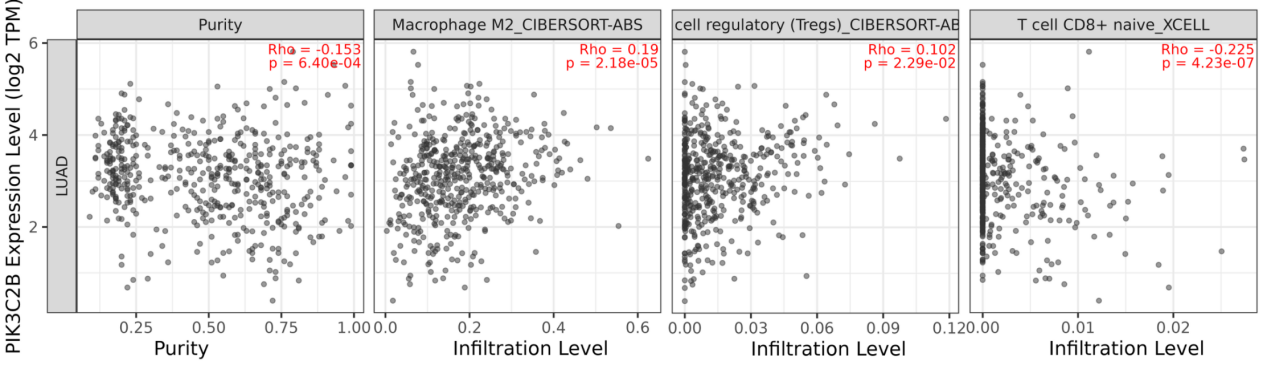


**Supplementary Figure 7**. We analyzed the correlation between PIK3C2B expression and immune cell infiltration levels using the TIMER2.0 database. PIK3C2B expression showed a significant positive correlation with the infiltration of M2 macrophages (Spearman r = 0.19, p < 0.001) and regulatory T cells (Tregs) (Spearman r = 0.102, p < 0.05), and a negative correlation with CD8+ T cells (Spearman r = -0.225, p < 0.001).


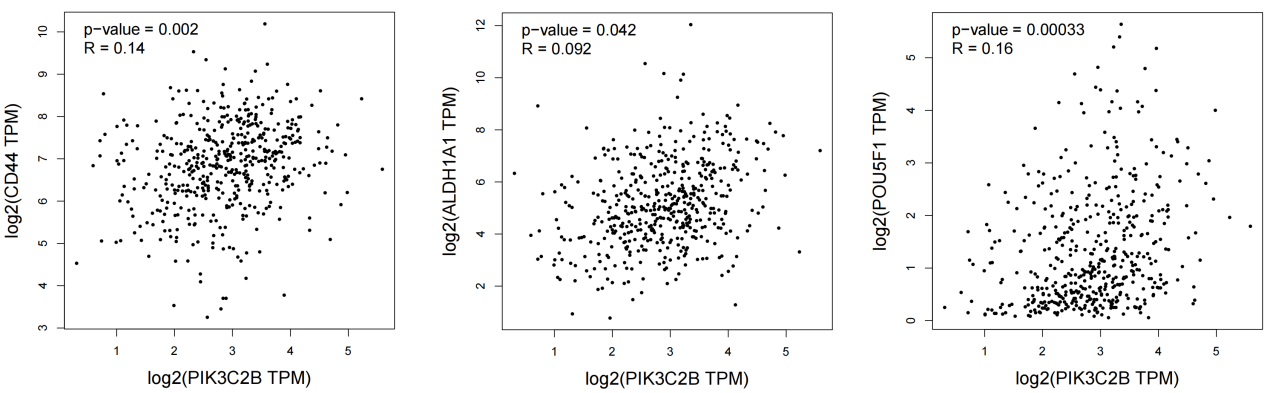


**Supplementary Figure 8**. The correlation of PIK3C2B expression with the expression of established lung cancer stemness markers such as CD44 (r=0.14, p<0.01), ALDH1A1 (r=0.092, p<0.05), and POU5F1 (OCT4, r=0.16, p<0.001) were analyzed via GEPIA database.


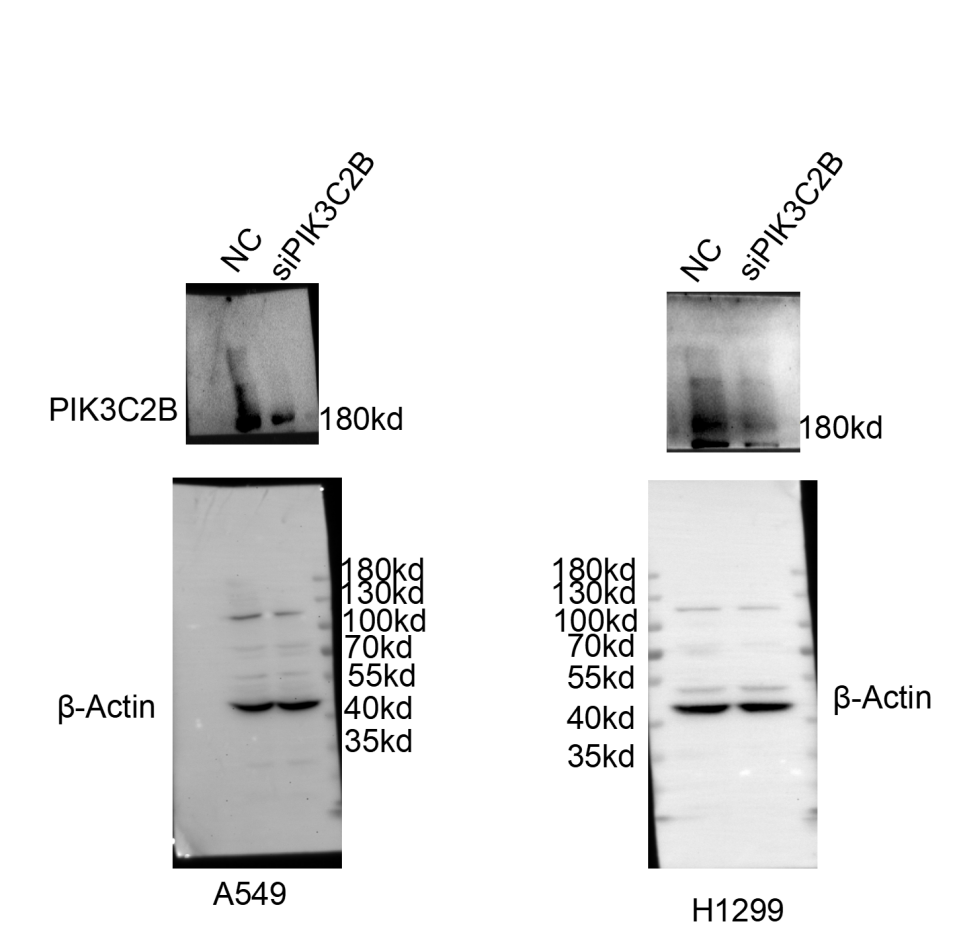


**Original figures for supplementary Figure 2**.


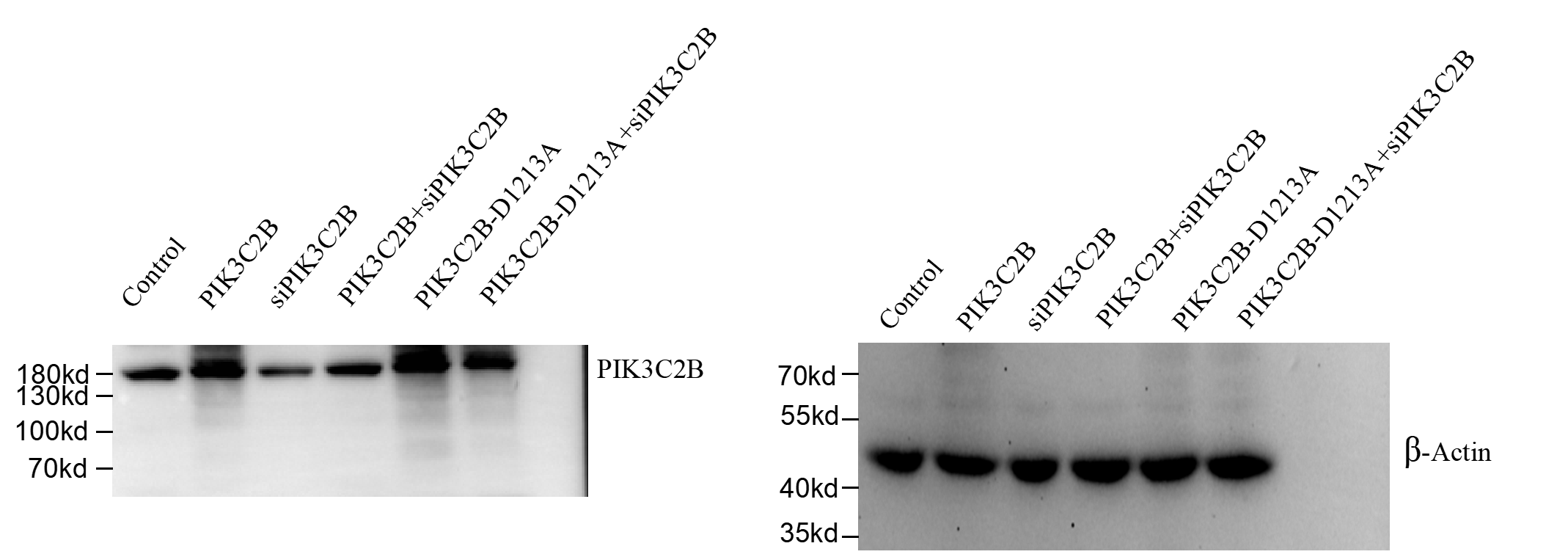


**Original figures for supplementary Figure 4**.
