# Supplementary figures and images for: PIK3C2B drives lung cancer progression through coordinating metabolic reprogramming and EMT-mediated metastasis
Source: Biochem Biophys Rep. 2025 Nov 21;44:102380. doi: 10.1016/j.bbrep.2025.102380 (PMC12681860; doi:10.1016/j.bbrep.2025.102380)

GAPDH

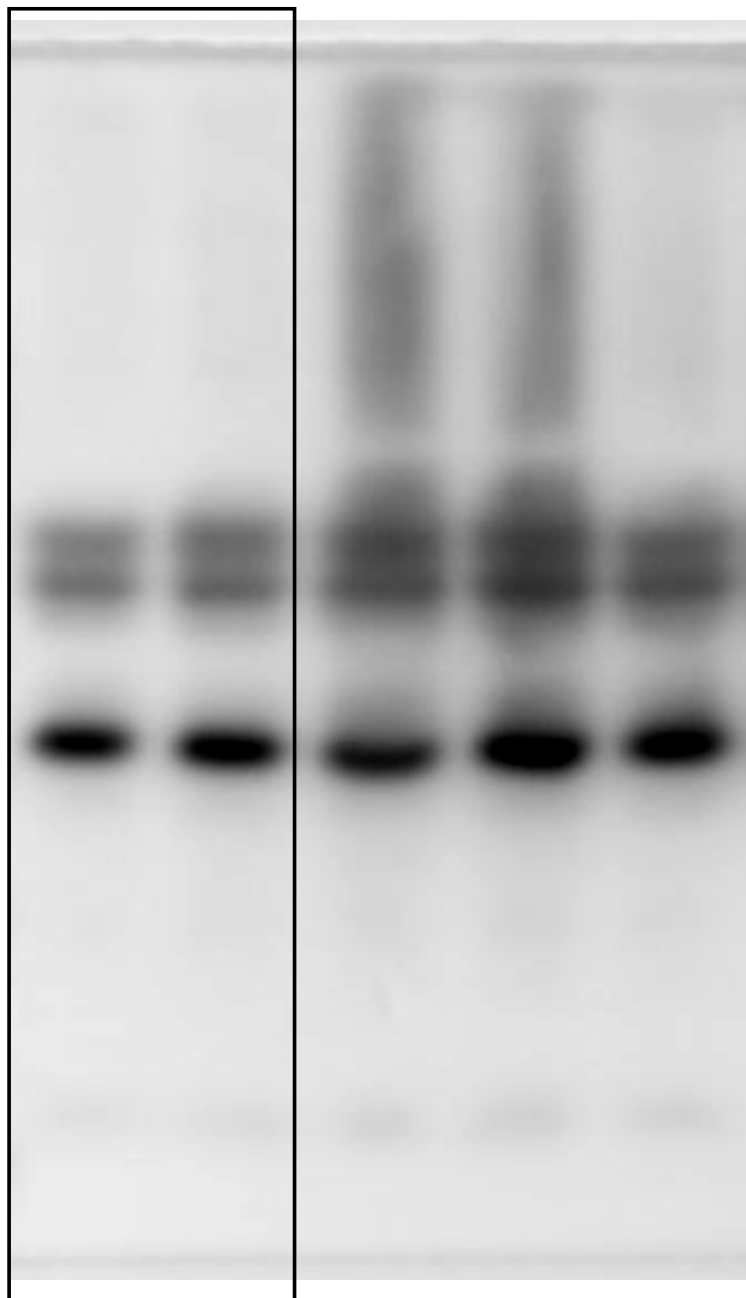

180kd

130kd

100kd

70kd

55kd

40kd

35kd

25kd

Supplement: Multimedia component 2 [file mmc2.pdf]
