# Supplementary material for: PIK3C2B drives lung cancer progression through coordinating metabolic reprogramming and EMT-mediated metastasis
Source: Biochem Biophys Rep. 2025 Nov 21;44:102380. doi: 10.1016/j.bbrep.2025.102380 (PMC12681860; doi:10.1016/j.bbrep.2025.102380)
Supplement: Multimedia component 3 [file mmc3.pdf]

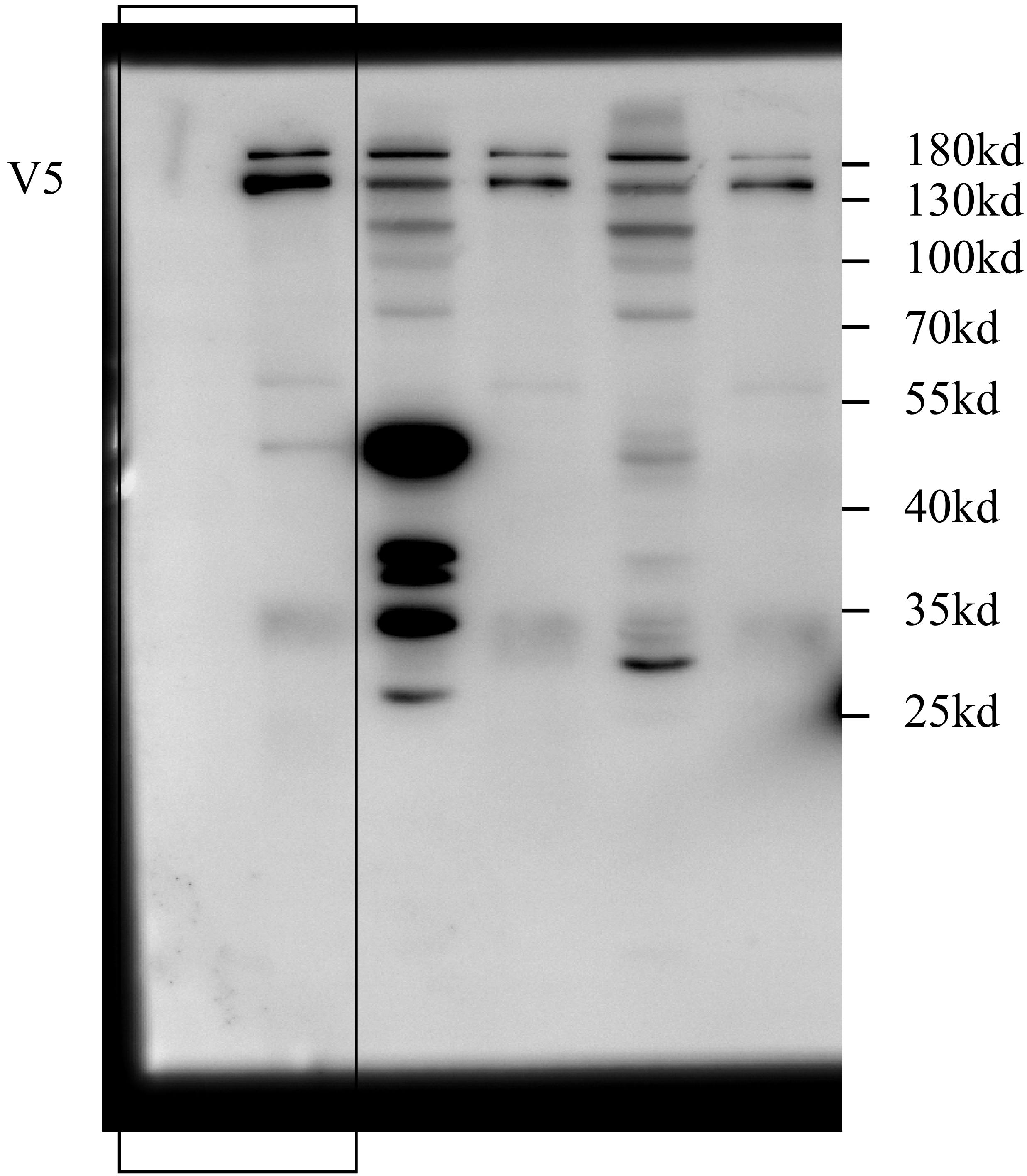

V5
180kd
130kd
100kd
70kd
55kd
40kd
35kd
25kd
